# Coumarins in Anticancer Therapy: Mechanisms of Action, Potential Applications and Research Perspectives

**DOI:** 10.3390/pharmaceutics17050595

**Published:** 2025-05-01

**Authors:** Tomasz Piotr Kubrak, Anna Makuch-Kocka, David Aebisher

**Affiliations:** 1 Department of Biochemistry and General Chemistry, Faculty of Medicine, Collegium Medicum, University of Rzeszow, 35-310 Rzeszów, Poland; 2 Department of Pharmacology, Faculty of Health Sciences, Medical University of Lublin, Radziwiłłowska 11 Street, 20-080 Lublin, Poland; anna.makuch-kocka@umlub.pl; 3 Department of Photomedicine and Physical Chemistry, Faculty of Medicine, Collegium Medicum, University of Rzeszow, 35-310 Rzeszów, Poland; daebisher@ur.edu.pl

**Keywords:** coumarins, anticancer activity of coumarins, in vivo studies, in vitro studies, pharmacokinetics of coumarins, bioavailability of coumarins

## Abstract

Coumarins are natural organic compounds widely found in plants that show promising anticancer properties. This article reviews the current research on the mechanisms of action of coumarins in cancer therapy, including the induction of apoptosis, inhibition of tumor cell proliferation, modulation of oxidative stress, and inhibition of angiogenesis and metastasis. Examples of coumarins with demonstrated anticancer activity, such as scopoletin, umbeliferon, esculetin and their synthetic derivatives, are also presented. The results of preclinical studies, the potential use of coumarins as stand-alone drugs and their role in combination therapy with chemotherapy are discussed. In addition, challenges related to bioavailability, safety and potential interactions with other drugs are highlighted. This review concludes by pointing out future research directions, such as the design of new coumarin analogs and the use of nanotechnology to enhance their efficacy in cancer treatment.

## 1. Introduction

**Figure 1:**
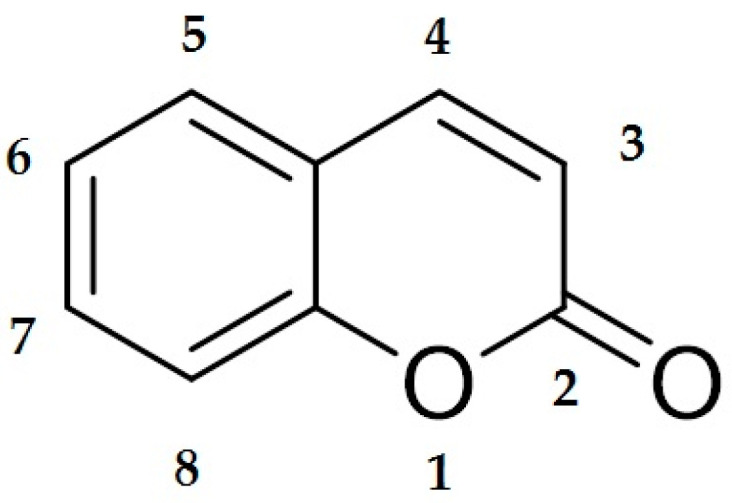
Chemical structure of coumarin. The illustration shows the basic skeleton of coumarin, consisting of a benzo-α-pyrone system in which the benzene ring is fused to the lactone ring of the α-pyrone. The numbering of the atoms in the molecule follows the convention used in organic chemistry. This structure is the basis for numerous coumarin derivatives with a wide range of biological and therapeutic properties.

Coumarins are a class of organic chemical compounds belonging to the benzopyrone lactone group, which are characterized by the presence of a 2H-1-benzopyran-2-one skeleton [1,2]. Their structure allows for constructing a wide range of derivatives that differ in their pharmacological activity (Figure 1).

**Figure 2:**
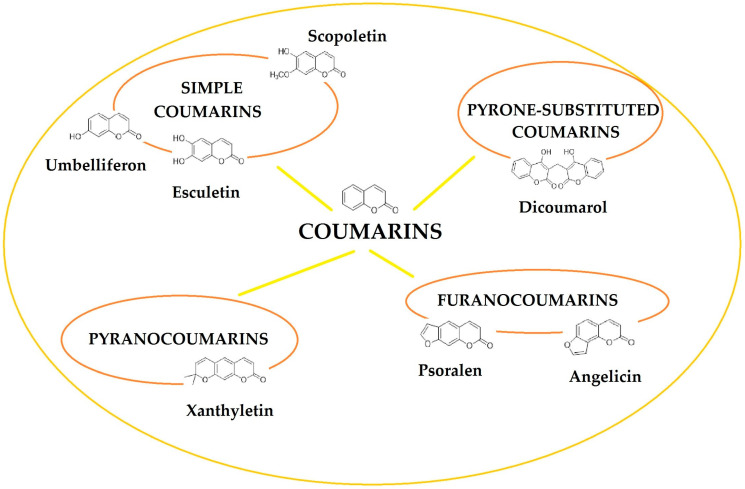
General classification of coumarins found in foods and plants.

Based on the chemical structure, coumarins can be divided into several groups, such as simple coumarins (e.g., umbeliferon, scopoletin, esculetin), furanocoumarins (e.g., psoralen, angelicin), pyranocoumarins (e.g., xanthyletin) and pyrone-substituted coumarins (e.g., dicoumarol). The scheme below shows the most important classes of coumarins (Figure 2).

Each of these groups has specific biological properties that can be exploited in medicine and pharmacology [3]. Coumarins have diverse properties and can function as antioxidants, antibacterial agents, anti-inflammatory agents and anticancer agents. It is this latter feature that is of growing interest to scientists investigating the potential applications of coumarins in oncology [4,5,6].

Coumarins occur naturally in plants of the *Apiaceae* (celery), *Fabaceae* (legumes), *Rutaceae* (rutaceae), and *Asteraceae* (daisy) families [7,8]. These compounds are synthesized by plants as secondary metabolites that are thought to act as protective agents against insects, fungi, and UV radiation. Natural sources of coumarins include horse chestnut (*Aesculus hippocastanum*), fennel (*Foeniculum vulgare*), angelica (*Angelica archangelica*), citrus fruits (*Citrus* spp.), chamomile (*Matricaria chamomilla*), and soybean (*Glycine max*) [9]. Some coumarins (e.g., psoralen and angelicin) form reactive intermediates upon exposure to UV radiation, which allows them to be used in PUVA photochemotherapy (psoralen + UVA), which is used in the treatment of psoriasis and some skin cancers [10].

The first reports of coumarins date back to the 19th century, when Vogel first isolated coumarin from tonka beans (*Dipteryx odorata*) in 1820, and in 1868, William Perkin synthesized it using the Perkin reaction [11]. In the 20th century, interest in coumarins increased after the discovery of their antithrombotic properties, which led to the development of warfarin, used as an anticoagulant drug [12]. In the following decades, research on coumarins began to focus on their potential use in anticancer therapy. Some natural and synthetic coumarins were found to have the ability to induce apoptosis, inhibit cancer cell proliferation, and inhibit angiogenesis [13,14,15]. In vitro and in vivo studies have confirmed the cytotoxic effects of coumarins, such as esculin, esculetin, scopoletin and umbeliferon, on different cancer cell lines [16,17,18,19]. Synthetic coumarins show even greater selectivity toward cancer cells, suggesting their potential use as modern anticancer drugs [3].

The aim of this article is to review the current research on the use of coumarins in anticancer therapy. The mechanisms of action of these compounds will be discussed, including their effect on apoptosis, cancer cell proliferation, oxidative stress and angiogenesis.

Preclinical and clinical studies will also be analyzed, as well as challenges related to the bioavailability and safety of coumarins. This article will also present future directions of research on new coumarins, especially in the context of the use of nanotechnology to improve their therapeutic efficacy. Due to the promising results of previous studies, coumarins may become a key element of modern oncological therapies in the future. Further research into them may lead to the development of more effective and selective anticancer drugs.

## 2. Mechanisms of Anticancer Action of Coumarins

**Figure 3:**
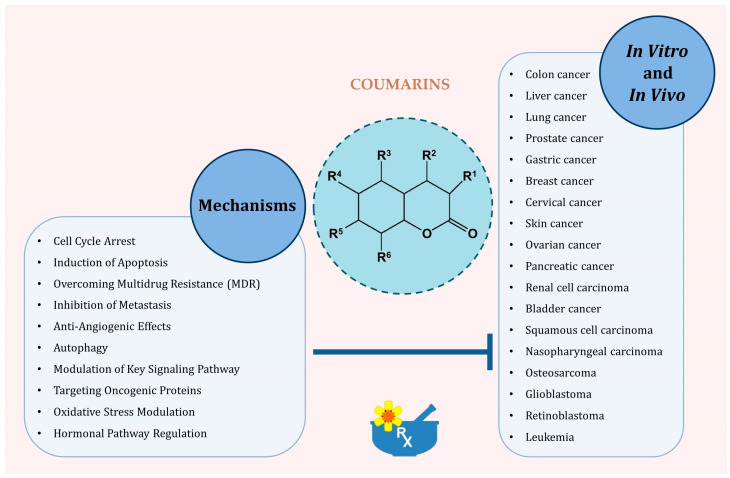
Principal molecular and cellular mechanisms through which coumarins exert antitumor effects.

Coumarin compounds exhibit multifaceted anticancer activity, including induction of cancer cell apoptosis, inhibition of proliferation and cell cycle, modulation of oxidative stress and antioxidant activity, and inhibition of angiogenesis and metastasis, as well as an influence on the immune system (Figure 3).

### 2.1. Induction of Apoptosis of Cancer Cells

Coumarins have shown promising anticancer properties, mainly due to their ability to induce apoptosis in cancer cells. Apoptosis, or programmed cell death, is a key mechanism that maintains homeostasis in the body and eliminates damaged or abnormal cells. In the context of cancer therapy, stimulating apoptosis in cancer cells is a promising therapeutic strategy.

Coumarins can initiate apoptosis by destabilizing the mitochondrial membrane, leading to the release of cytochrome c into the cytoplasm, which activates caspases responsible for cell degradation. Studies have shown that osthole, a natural furanocoumarin, induces apoptosis in cancer cells by activating caspases and modulating Bcl-2 and Bax proteins, which regulate mitochondrial membrane permeability [20,21,22]. Some coumarins (e.g., psoralidin) can interact with death receptors on the surface of cancer cells, such as TRAIL-R1/DR4 and TRAIL-R2/DR5. Activation of these receptors leads to a signaling cascade that results in apoptosis [23]. TRAIL (TNF-related apoptosis-inducing ligand) is a protein that induces apoptosis in cancer cells, with low toxicity to normal cells [24]. Coumarins can affect the activity of proteins such as p53, known as the “guardian of the genome”. Activation of p53 leads to cell cycle arrest and induction of apoptosis in cells with damaged DNA. Mutations in the p53 gene are frequently observed in cancers, and modulation of its activity by coumarins may restore the ability of cells to undergo programmed death [25,26].

Cancer cells often show excessive activity of pro-survival signaling pathways such as PI3K/Akt/mTOR [27]. Coumarins can inhibit these pathways, leading to reduced proliferation and induction of apoptosis. Osthole, for example, inhibits cancer cell proliferation by arresting the cell cycle and inducing apoptosis, which may be related to modulation of the PI3K/Akt/mTOR pathway [28].

Coumarins exhibit multifaceted anticancer activity by inducing apoptosis in cancer cells. Their ability to modulate key signaling pathways and proteins involved in cell survival processes makes them promising candidates in anticancer therapy. Further studies on the mechanisms of their action may contribute to the development of new strategies for cancer treatment.

### 2.2. Inhibition of Cell Proliferation and Cycle

One of the key mechanisms of action is the inhibition of cancer cell proliferation and modulation of the cell cycle. Cancer cell proliferation is a process of uncontrolled cell division leading to the development and progression of cancer. Studies have shown that some coumarins can effectively inhibit this process. For example, esculetin (6,7-dihydroxycoumarin) inhibits cell growth and progression by inducing G1 arrest in human HL-60 leukemia cells. This mechanism involves an increase in the unphosphorylated retinoblastoma protein (pRb) and a decrease in the CDK4 levels, as well as an upregulation of p27 and a downregulation of cyclin D1. The results suggest that by inhibiting pRb phosphorylation, esculetin may arrest the cell cycle in G1, leading to the inhibition of HL-60 leukemia cell growth [29]. In another study, isoimperatorin (ISOIM), a natural furanocoumarin, showed the ability to inhibit the proliferation of gastric cancer cells by inducing cell cycle arrest in the G2/M phase. Additionally, ISOIM induced apoptosis by increasing Bax expression and decreasing Bcl-2 expression, leading to a decreased Bcl-2/Bax ratio compared with control cells [30].

The cell cycle consists of a series of ordered steps leading to cell division. In cancer cells, the regulation of this cycle is often disrupted, which promotes their uncontrolled proliferation. Coumarins can intervene in this process, leading to cell cycle arrest at specific phases. Studies have shown that 7-hydroxycoumarin has a more potent cytostatic effect than coumarin in the human adenocarcinoma cell line A427, which has a positive pRb and homozygous deletion in the p16^INK4a^ gene. Inhibition of the cell cycle at the G1/S transition is consistent with the cytostatic effect of 7-hydroxycoumarin and does not cause changes in the level of cyclin D1 mRNA after transcription [31].

In summary, coumarins show potential as anticancer compounds by inhibiting cancer cell proliferation and modulating the cell cycle. These mechanisms include induction of cell cycle arrest at specific phases and regulation of apoptosis-related proteins, which limits the ability of cancer cells to divide uncontrolled. Further studies on these compounds may contribute to the development of new therapeutic strategies for cancer treatment.

### 2.3. Modulation of Oxidative Stress and Antioxidant Effects

A pivotal mechanism underlying the anticancer effects of coumarin is the modulation of oxidative stress due to their inherent antioxidant activity. Oxidative stress arises from an imbalance between the production of reactive oxygen species (ROS) and the body’s ability to neutralize them. Elevated ROS levels can inflict damage to DNA, proteins, and lipids, thereby promoting carcinogenesis. Consequently, modulating oxidative stress is a strategic approach in cancer prevention and therapy.

Coumarins, which are heterocyclic compounds, have been shown to have a number of beneficial health effects, including reducing the risk of cancer, diabetes, and cardiovascular and brain diseases. These properties are attributed to their ability to neutralize free radicals through antioxidant activity [32]. Studies have shown that both natural and synthetic coumarin derivatives can significantly affect the functioning of various mammalian cellular systems. Their development as potential antioxidant agents has attracted the attention of scientists due to their unique mechanisms of action [33]. Interestingly, even non-phenolic coumarin derivatives, such as 7-dialkyl-aminocoumarins, have significant antioxidant properties. This mechanism is associated with the presence of trace amounts of reactive compounds such as hydroxycinnamic acid, which can neutralize free radicals. It is also worth noting that structural modifications of the coumarin molecule can affect its antioxidant activity. Changes in the C-3 and C-4 positions can shift the reactivity of the compounds toward different types of free radicals, suggesting the possibility of designing more effective antioxidants based on the coumarin structure [34].

Numerous studies have demonstrated that coumarins possess significant antioxidant capabilities. These compounds can scavenge ROS and inhibit oxidative processes, thereby protecting cells from oxidative damage. For instance, certain coumarin derivatives have been shown to suppress oxidative stress through their ability to scavenge ROS and inhibit neutrophil-dependent superoxide anion formation. Additionally, coumarins can modulate key signaling pathways associated with oxidative stress responses. The Keap1/Nrf2/ARE pathway plays a crucial role in cellular defense against oxidative stress by regulating the expression of antioxidant proteins. Coumarins have been identified as modulators of this pathway, enhancing the transcription of genes involved in antioxidant defense [35].

Interestingly, especially at higher concentrations, coumarins can exhibit pro-oxidant properties, leading to increased ROS production. This controlled elevation in ROS can trigger apoptosis in cancer cells. For example, 4-phenylcoumarin has been reported to induce ROS-dependent cell death in A549 lung cancer cells, highlighting its potential as a therapeutic agent [36].

The dual ability of coumarins to act as antioxidants and, under specific circumstances, as pro-oxidants underscores their potential in cancer therapy. By modulating oxidative stress, coumarins can protect normal cells from damage and selectively induce death in cancer cells. Further research into these mechanisms may pave the way for the development of coumarin-based anticancer therapeutics.

### 2.4. Inhibition of Angiogenesis and Metastasis

Coumarins, a class of naturally occurring benzopyrone compounds, have demonstrated significant anticancer properties, notably through the inhibition of angiogenesis and metastasis. These processes are critical for tumor growth and dissemination, and targeting them is a promising therapeutic strategy [37].

Angiogenesis, the formation of new blood vessels from existing vasculature, is essential for tumor progression. Coumarins have been shown to inhibit angiogenesis through various mechanisms.

Modulation of VEGF signaling: Vascular endothelial growth factor (VEGF) is a primary mediator of angiogenesis. Coumarins can suppress VEGF expression and its receptor activity, thereby hindering endothelial cell proliferation and new vessel formation. For instance, studies have demonstrated that coumarin derivatives effectively reduce VEGF-induced angiogenesis in cancer models [14,38].

Suppression of endothelial cell functions: Coumarins directly affect endothelial cells by inhibiting their proliferation, migration, and tube formation, which are vital steps in angiogenesis. Research indicates that certain coumarin compounds disrupt endothelial cell functions, leading to impaired angiogenic processes [39].

Metastasis, the spread of cancer cells to distant organs, is a leading cause of cancer-related mortality. Coumarins have been found to impede metastasis through several pathways.

Inhibition of matrix metalloproteinases (MMPs): MMPs degrade extracellular matrix components, facilitating cancer cell invasion. Coumarins have been shown to inhibit metalloproteinase-2 (MMP-2) and metalloproteinase-9 (MMP-9) activity, thereby reducing tumor cell migration and invasion [40]. 

Suppression of epithelial–mesenchymal transition (EMT): EMT is a process where epithelial cells acquire mesenchymal properties, enhancing migratory and invasive capabilities. Coumarins can inhibit EMT by modulating key signaling pathways, thus reducing the metastatic potential [41,42,43].

The ability of coumarins to inhibit angiogenesis and metastasis underscores their potential as therapeutic agents in cancer treatment. By targeting multiple pathways involved in tumor vascularization and dissemination, coumarins offer a multifaceted approach to impeding cancer progression.

### 2.5. Impact on the Immune System

Coumarins have demonstrated significant immunomodulatory properties, impacting both innate and adaptive immune responses.

Research has shown that coumarin derivatives can enhance T-cell proliferation and activity. For instance, a study Berkarda et al. observed increased lymphocyte responsiveness to phytohemagglutinin (PHA) in both normal individuals and cancer patients treated with coumarin derivatives [44]. Coumarins influence the production of key cytokines involved in inflammatory responses. A systematic review highlighted that coumarins act on immune cells and cytokines, playing a role in the treatment of autoimmune diseases by regulating signaling pathways such as NF-κB and Keap1/Nrf2. The NF-κB pathway is pivotal in regulating immune responses and inflammation. Coumarins have been found to inhibit NF-κB activation, leading to decreased expression of inflammatory mediators. For example, studies have shown that coumarins can modulate this pathway, thereby exhibiting anti-inflammatory effects. Keap1/Nrf2 Pathway: The Keap1/Nrf2 pathway plays a crucial role in the cellular defense against oxidative stress. Coumarins can activate the Nrf2 pathway, enhancing the expression of antioxidant proteins and contributing to the modulation of immune responses. This mechanism has been highlighted in studies exploring the therapeutic potential of coumarins in autoimmune diseases [35,45].

Due to their immunomodulatory properties, coumarins have been explored as potential therapeutic agents in autoimmune diseases. A comprehensive review discussed the therapeutic effects of natural coumarins in autoimmune diseases, emphasizing their role in modulating immune responses [45].

The therapeutic potential of coumarins in autoimmune diseases has been highlighted in various studies, suggesting their role in modulating immune responses and providing a basis for the development of new treatments. For instance, a study demonstrated that coumarin derivatives ameliorate intestinal inflammation and modulate the gut microbiome, highlighting their potential in treating autoimmune conditions [46].

Coumarins exert significant effects on the immune system by modulating immune cell functions, cytokine production, and key signaling pathways. These properties underscore their potential as therapeutic agents in managing immune-related disorders, including autoimmune diseases and inflammatory conditions.

## 3. Coumarins with Proven Anticancer Effects

### 3.1. Scopoletin and Its Biological Activity

**Figure 4:**
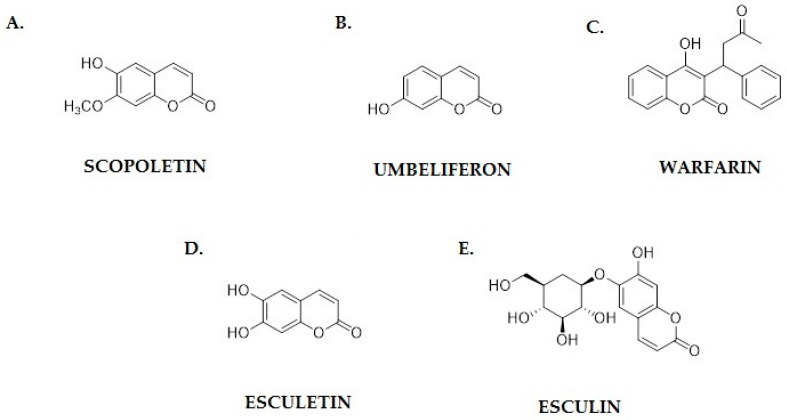
Chemical structures of coumarins with proven anticancer activity: (**A**) scopoletin; (**B**) umbeliferon; (**C**) warfarin; (**D**) esculetin; and (**E**) esculin.

Scopoletin (Figure 4A), a member of the coumarins group, is a natural chemical compound found in many plant species. Numerous studies indicate its broad spectrum of biological activity, including anti-inflammatory, antioxidant and antimicrobial effects [47].

In recent years, there has been growing interest in its anticancer potential, especially in the context of various types of cancers, such as cervical cancer, gynecological cancers and other types of solid tumors. One of the key mechanisms of scopoletin’s anticancer action is the induction of apoptosis in cancer cells. Studies conducted by Karatoprak et al. have shown that scopoletin, as a representative of coumarins, can act as a multi-target therapeutic anticancer agent by activating apoptotic pathways in gynecological cancer cells. The results of in vitro studies on cervical cancer cell lines (HeLa) suggest that this compound leads to DNA fragmentation and increases caspase activity [48,49]. Scopoletin also has antioxidant activity, which may play an important role in inhibiting the growth of cancer cells. Ajiboye et al. analyzed plant extracts containing scopoletin and showed that this substance can reduce oxidative stress in cells, which leads to a reduction in cancer proliferation. An increase in the level of reactive oxygen species (ROS) in cancer cells can lead to their death, and scopoletin modulates this process [47]. Studies by Meng et al. showed that scopoletin affects the biosynthetic pathways of coumarins and the mechanisms regulating the proliferation and apoptosis of cancer cells. Their experiments, conducted on cell cultures, showed that the presence of scopoletin leads to the inhibition of the expression of genes responsible for the development of cancer, such as Bcl-2 and c-Myc [50].

In addition to its direct effect on cancer cells, scopoletin may also affect the tumor microenvironment. The work of Elgabry et al. suggests that this compound may modify the activity of the immune system, strengthening the body’s immune response to cancer cells [51]. In turn, the review by Sun and Shahrajabian indicated a synergistic effect of scopoletin with other plant-derived compounds that support its therapeutic properties. Due to its properties, scopoletin is considered a potential component of anticancer drugs. It can be used as a stand-alone active substance or in combination with other chemotherapeutic drugs to increase the effectiveness of therapy and reduce the side effects of traditional chemotherapy [52]. The work suggests that its combination with other coumarins may additionally enhance the therapeutic effect.

Scopoletin exerts strong anticancer properties by inducing apoptosis, inhibiting proliferation, modulating oxidative stress, and influencing the tumor microenvironment. Its potential use in anticancer therapy requires further clinical trials to confirm its efficacy and safety. The results of the studies to date are promising and indicate the possibility of using scopoletin as an innovative component of cancer therapy.

### 3.2. Umbeliferon—Mechanisms of Anticancer Action

Umbeliferon (7-hydroxycoumarin; Figure 4B) is a natural phenolic compound belonging to the coumarins group, which exhibits a wide range of biological activities, including anti-inflammatory, antioxidant and antimicrobial activities. In recent years, there has been growing interest in its anticancer potential, resulting from its ability to modulate various signaling pathways important for the proliferation, apoptosis and migration of cancer cells [53]. One of the key mechanisms of action of umbeliferon is the ability to induce apoptosis in cancer cells. Studies by Albratty and Makeen have shown that umbeliferon can reduce the expression of the Bcl-2 protein, which plays a key role in inhibiting apoptosis, while increasing the expression of proapoptotic Bax. As a result, caspases are activated and cancer cells die, which suggests the therapeutic potential of this compound in the treatment of breast cancer [54]. Similar results were obtained by Muthu et al. when examining the effect of umbeliferon on colon cancer cells. Their experiments showed that this compound activates the caspase cascade and increases the cytochrome c levels in the cytoplasm, leading to apoptosis [55].

The mechanism of action of umbeliferon also includes modulation of oxidative stress in cancer cells. Yu et al. demonstrated that in liver cancer, umbeliferon increases the reactive oxygen species (ROS) levels, which causes DNA damage and activation of the p53 pathway. This leads to cell cycle arrest, and ultimately, to cancer cell death [56].

Furthermore, it has been shown that umbeliferon can act as both an antioxidant and a pro-oxidant depending on the biological context. In healthy cells, it counteracts oxidative stress, while in cancer cells, it leads to increased ROS production and apoptosis [57].

The growth and migration of cancer cells are key processes leading to the development of malignant tumors. Ma et al. showed that umbeliferon inhibits glioma cell proliferation and metastasis by regulating the cadherin/β-catenin complex. Reduced activity of this complex leads to decreased intercellular adhesion and inhibition of the invasive properties of cancer cells [58]. Similar effects were reported by Lim et al. in the context of skin disease. Their studies showed that umbeliferon inhibits NF-κB activity, which results in reduced expression of pro-inflammatory factors and reduced ability of the tumor to evade the immune system [59].

Umbeliferon has the ability to affect many signaling pathways crucial to cancer cell survival. It has been proven that this compound blocks the PI3K/Akt/mTOR pathway, which is overactivated in many types of cancer. Inhibition of this pathway leads to cell cycle arrest in the G1 phase and reduced cancer cell survival [53,60]. In turn, An et al. showed that umbeliferon can also affect the MAPK/ERK pathway, which plays an important role in regulating cell proliferation and differentiation. Their studies suggest that umbeliferon may be a promising compound for targeted therapy [61].

Due to its multidirectional anticancer activity, umbeliferon is considered as a potential component of combination therapies [62]. It can be used in combination with other chemotherapeutic drugs to increase their efficacy and reduce cancer’s resistance to treatment. Moreover, its natural origin and relatively low toxicity make it an attractive candidate for clinical trials.

### 3.3. Warfarin and Its Role in Inhibiting Angiogenesis

Angiogenesis, the process of new blood vessel formation, is crucial for various physiological and pathological processes, including wound healing, embryogenesis, and cancer progression. The regulation of angiogenesis is mediated by a complex interplay of growth factors, signaling pathways, and extracellular matrix remodeling [63]. Targeting angiogenesis has become a prominent strategy in cancer therapy and the treatment of diseases characterized by excessive vascularization, such as diabetic retinopathy [64].

Warfarin (Figure 4C), a widely used anticoagulant, has recently been recognized for its potential antiangiogenic properties. It functions primarily as a vitamin K antagonist, interfering with the γ-carboxylation of coagulation proteins. However, research suggests that its effects extend beyond anticoagulation, influencing angiogenesis by modulating vascular endothelial growth factor (VEGF) signaling, endothelial cell migration, and tumor microenvironment interactions [65].

VEGF is a key regulator of angiogenesis, promoting endothelial cell proliferation and new vessel formation. Warfarin has been shown to interfere with VEGF-driven angiogenesis by disrupting Gla-domain protein activation, which is essential for endothelial function [66,67]. In vitro studies demonstrate that warfarin inhibits VEGF-induced endothelial cell migration and tube formation, leading to the suppression of neovascularization [68]. Additionally, warfarin modulates the expression of VEGF receptors, particularly VEGFR-2, reducing its downstream signaling and limiting the angiogenic response [69]. These findings suggest that warfarin’s inhibitory effects on angiogenesis are mediated through direct interaction with key proangiogenic pathways.

Endothelial cell migration and tube formation are essential steps in angiogenesis. Studies indicate that warfarin suppresses endothelial proliferation by interfering with protease-activated receptor 1 (PAR-1) signaling, which plays a vital role in vascular remodeling [70]. Experimental models reveal that warfarin-treated endothelial cells exhibit reduced motility and an impaired ability to form capillary-like structures, further supporting its antiangiogenic potential.

Furthermore, warfarin has been found to disrupt hypoxia-induced angiogenesis, a key mechanism in tumor vascularization. Hypoxic conditions stimulate the expression of hypoxia-inducible factor-1α (HIF-1α), which in turn upregulates VEGF production. Warfarin counteracts this process by downregulating HIF-1α activity, thereby attenuating the hypoxia-driven angiogenic response [71].

Tumor growth and metastasis depend on the formation of new blood vessels to supply oxygen and nutrients. Warfarin has been shown to inhibit tumor-associated angiogenesis by targeting multiple signaling pathways involved in endothelial activation and vascular remodeling [63]. Studies on cancer models reveal that warfarin, in combination with chemotherapeutic agents, enhances treatment efficacy by reducing the vascular density within tumors and limiting the supply of essential nutrients for tumor progression. Moreover, warfarin’s ability to modulate the tumor microenvironment suggests its potential as an adjuvant therapy in cancer treatment [72].

The antiangiogenic properties of warfarin highlight its potential for repurposing as an adjunctive therapy in cancer treatment. Despite promising preclinical findings, clinical studies are needed to determine the optimal dosing and minimize the potential side effects, particularly the risk of hemorrhage associated with long-term anticoagulant use.

### 3.4. Esculetin and Esculin in Cancer Models

Esculetin and esculin (Figure 4D,E) are naturally occurring coumarin derivatives that have attracted significant attention due to their anticancer properties. These compounds exhibit a wide range of biological activities, including antioxidant, anti-inflammatory, and antiproliferative effects, making them promising candidates for cancer treatment [15,73]. Studies indicate that esculetin, the aglycone form of esculin, plays a crucial role in modulating key cellular pathways involved in tumor progression, apoptosis, and metastasis [74].

One of the primary anticancer mechanisms of esculetin and esculin is their ability to induce apoptosis in cancer cells. Studies have demonstrated that esculetin triggers apoptosis through the activation of caspase-dependent pathways and mitochondrial dysfunction, leading to the release of cytochrome c and subsequent cell death [15,16]. Additionally, esculetin has been shown to cause G1 and G2/M cell cycle arrest, preventing uncontrolled proliferation of cancer cells [73]. Tumor progression relies heavily on angiogenesis, the formation of new blood vessels, and metastasis, the spread of cancer cells to distant organs. Esculetin exhibits strong antiangiogenic and antimetastatic properties by downregulating vascular endothelial growth factor (VEGF) and matrix metalloproteinases (MMPs), which are critical regulators of cancer cell invasion and migration [35]. Furthermore, in melanoma models, esculetin has demonstrated synergistic effects when combined with conventional chemotherapeutics, enhancing their efficacy in reducing the metastatic potential [75]. Oxidative stress plays a crucial role in carcinogenesis by causing DNA damage, promoting tumor growth, and enhancing drug resistance. Esculin and esculetin exhibit potent antioxidant properties by scavenging reactive oxygen species (ROS) and modulating nuclear factor erythroid 2-related factor 2 (Nrf2) signaling, which regulates cellular antioxidant defense mechanisms [35]. Moreover, these compounds have been found to suppress inflammatory pathways by inhibiting NF-κB activation, which is implicated in tumor initiation and progression [16].

In breast cancer models, esculetin has been found to inhibit the proliferation of estrogen receptor-positive by modulating estrogen receptor signaling and inducing autophagy-related cell death [76]. Furthermore, studies suggest that combining esculetin with standard chemotherapeutic agents enhances treatment outcomes by reducing drug resistance mechanisms [73]. Colorectal cancer (CRC) is one of the most common malignancies worldwide. Esculin and esculetin have shown promising anti-CRC activity by targeting key oncogenic pathways such as Wnt/β-catenin and PI3K/Akt/mTOR signaling [77,78]. Studies also report that esculetin sensitizes CRC cells to radiotherapy and chemotherapy, thereby improving treatment efficacy and reducing tumor recurrence rates [79].

In melanoma models, esculetin has been investigated for its ability to suppress tumor growth and enhance the effects of existing anti-melanoma drugs. Research suggests that esculetin exerts its effects by modulating oxidative stress responses and inhibiting melanogenesis-related proteins. Isobolographic analysis has revealed that esculetin displays synergistic interactions with doxorubicin and cisplatin, leading to enhanced cytotoxic effects in melanoma cells [75].

Gastrointestinal cancers, including gastric and pancreatic cancer, are highly aggressive malignancies with a poor prognosis. Studies indicate that esculetin inhibits tumor growth by downregulating pro-inflammatory cytokines, which contribute to the inflammatory tumor microenvironment. Additionally, esculetin has been shown to target ATP-binding cassette (ABC) transporters, which are responsible for multidrug resistance in gastrointestinal tumors [80].

Despite promising preclinical data, the clinical application of esculetin and esculin remains limited due to challenges related to bioavailability and pharmacokinetics. Studies are currently underway to develop novel drug delivery systems, such as nanoformulations and encapsulation techniques, to improve their stability and therapeutic efficacy [16]. Moreover, ongoing clinical trials are investigating the potential of esculetin as an adjuvant therapy in combination with conventional anticancer agents [81].

Future research should focus on elucidating the molecular targets of esculetin and esculin in different cancer subtypes, optimizing their pharmacological profiles, and conducting large-scale clinical trials to establish their safety and efficacy in human patients [82].

Esculetin and esculin have emerged as promising natural compounds with significant anticancer potential. Their ability to induce apoptosis, inhibit angiogenesis, and modulate oxidative stress makes them attractive candidates for cancer therapy. While current studies provide strong preclinical evidence supporting their efficacy, further research is needed to overcome the pharmacokinetic limitations and translate these findings into clinical applications. The development of novel drug formulations and combination strategies could pave the way for integrating esculetin and esculin into standard cancer treatments.

### 3.5. New Synthetic Coumarins with Therapeutic Potential

In recent years, advancements in synthetic chemistry have led to the development of novel coumarin derivatives with enhanced therapeutic potential [83]. These synthetic coumarins have demonstrated improved bioavailability, specificity, and efficacy against various diseases, particularly in cancer therapy [39].

One of the most significant advancements in synthetic coumarins is the introduction of four-substituted derivatives, which have shown promising anticancer properties. These compounds exhibit potent cytotoxic effects against cancer cells by targeting specific molecular pathways, such as the inhibition of topoisomerases and modulation of apoptosis-related proteins [84]. Sulfonamide-modified coumarins have gained attention due to their ability to inhibit key enzymes involved in cancer progression. Recent studies have shown that these derivatives act as selective enzyme inhibitors, effectively suppressing tumor growth while minimizing side effects [85]. Hybridization of coumarins with other pharmacophores has led to the development of compounds with enhanced selectivity and potency. Recent research highlights the potential of hybrid coumarins in photodynamic therapy, where they serve as mitochondrial-targeted photosensitizers in anticancer treatment [86].

Synthetic coumarins exert their anticancer effects through multiple mechanisms, with apoptosis induction being a primary mode of action. Many newly developed coumarin derivatives activate intrinsic and extrinsic apoptotic pathways by upregulating pro-apoptotic proteins and downregulating anti-apoptotic proteins [87]. Coumarin-based derivatives have been shown to halt cancer cell proliferation by inducing cell cycle arrest at different phases. Studies indicate that specific coumarins can effectively inhibit cyclin-dependent kinases (CDKs), leading to the accumulation of cells in the G1 or G2/M phase, thereby preventing their progression into mitosis [15,88].

**Figure 5:**
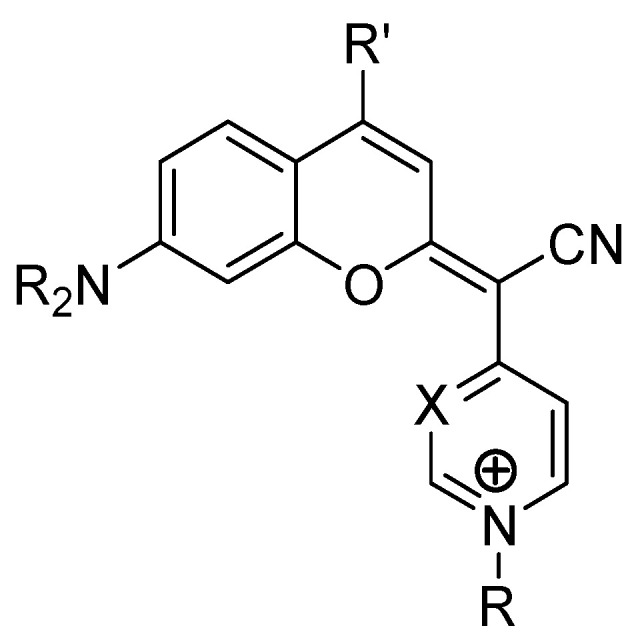
Chemical structure of coumarin-based COUPY derivatives.

A recent breakthrough in coumarin-based therapy is their application in photodynamic therapy (PDT). COUPY coumarins (Figure 5), for instance, have been engineered to selectively accumulate in mitochondria, enhancing their photodynamic effects and selectively eliminating cancer cells [86]. This targeted approach minimizes the damage to healthy tissues, making PDT a promising strategy in modern oncology.

Breast cancer remains one of the most studied malignancies for coumarin-based therapies. Synthetic coumarins have demonstrated potent effects in hormone-sensitive and triple-negative breast cancer models by inhibiting estrogen receptor signaling and inducing autophagic cell death [89]. Lung cancer is another area where synthetic coumarins show great promise. Research indicates that modified coumarin derivatives can effectively suppress key oncogenic pathways, such as PI3K/Akt and MAPK, leading to significant tumor growth inhibition [90]. Colorectal cancer (CRC) is among the leading causes of cancer-related mortality worldwide. Novel coumarins have shown efficacy in CRC models by downregulating β-catenin signaling and inhibiting multidrug resistance proteins [91].

4-Hydroxycoumarin (4-HC) and its derivatives have attracted increasing attention in recent years due to their promising anticancer activities. Structurally, 4-HC serves as a core scaffold for the synthesis of numerous biologically active molecules capable of interacting with key cellular targets involved in tumor progression. Recent research by Dimić et al. (2022) demonstrated that novel derivatives of 4-hydroxycoumarin conjugated with neurotransmitters such as dopamine and octopamine exhibit significant selective cytotoxicity toward colorectal carcinoma (HCT-116) and cervical adenocarcinoma (HeLa) cells, while maintaining low toxicity toward healthy fibroblast cells (MRC-5). The proposed mechanisms underlying the observed anticancer effects include the induction of apoptosis, inhibition of carbonic anhydrase IX (hCA-IX)—a marker of tumor hypoxia—and modulation of oxidative stress through altered reactive oxygen species (ROS) production. Molecular docking and dynamics studies confirmed the strong interactions of these derivatives with hCA-IX, supporting their potential as selective anticancer agents [92]. Additionally, Avdović et al. (2021) reported the synthesis and biological evaluation of new 4-hydroxycoumarin-based ligands and their palladium(II) complexes. The study revealed that these complexes possess enhanced cytotoxic properties against several carcinoma cell lines, including colorectal (HCT116), melanoma (A375), and pancreatic carcinoma (MIA PaCa-2) cells. Notably, the cytotoxicity was primarily mediated through apoptosis induction. The palladium(II) complexes exhibited higher inhibitory activity compared to their parent ligands, suggesting that metal complexation significantly improves the anticancer potential of 4-hydroxycoumarin scaffolds [93].

Both studies emphasized that modifications to the basic 4-HC structure, such as the attachment of neurotransmitter fragments or complexation with metal ions, can optimize pharmacological profiles, enhance selectivity, and reduce toxicity toward healthy tissues. Importantly, ecotoxicological assessments indicated that these new derivatives have a low environmental impact, further supporting their potential in clinical applications.

Despite the promising therapeutic potential of synthetic coumarins, several challenges remain in their clinical translation. Issues such as poor solubility, rapid metabolism, and potential toxicity must be addressed through advanced drug formulation techniques, such as nanoparticle-based delivery systems [94]. Moreover, further research is needed to explore their combinatorial effects with standard chemotherapeutics to enhance their efficacy while reducing their side effects [95].

The development of synthetic coumarins represents a significant advancement in medicinal chemistry, particularly in cancer therapy. These novel compounds exhibit potent anticancer properties through multiple mechanisms, including apoptosis induction, cell cycle arrest, and targeted photodynamic therapy. While challenges in terms of bioavailability and toxicity remain, continued research and technological innovations hold the potential to establish synthetic coumarins as viable therapeutic agents in modern oncology.

## 4. Research Models and Results of Preclinical Studies

Coumarins, a prominent class of naturally occurring and synthetically modified benzopyrones, have gained considerable attention in anticancer research due to their potent bioactivities. Preclinical studies, encompassing in vitro, in vivo, and pharmacokinetic analyses, play a critical role in evaluating the efficacy, mechanisms of action, and potential clinical applications of these compounds. This section examines recent advancements in coumarin-based preclinical research models, providing an in-depth overview of their impact on cancer cell lines, animal models, and pharmacokinetic properties.

### 4.1. In Vitro Studies—The Effect of Coumarins on Various Cancer Cell Lines

Coumarin derivatives exhibit significant cytotoxic and pro-apoptotic effects across various cancer cell lines. Recent research has shown that *0*-prenylated coumarin derivatives induce cell death in human cervical cancer cells by activating caspase-dependent apoptosis [96]. Similar effects have been observed in melanoma, where daphnetin, a hydroxylated coumarin, enhances chemosensitivity and potentiates the activity of standard chemotherapy agents [97]. Moreover, novel coumarin-6-sulfonamides have been identified as potent apoptotic antiproliferative agents. These derivatives demonstrate strong inhibition of cell viability in colorectal and breast cancer cell lines by downregulating Bcl-2 and upregulating Bax expression [98].

Beyond direct cytotoxicity, coumarins also exhibit antiangiogenic properties by suppressing vascular endothelial growth factor (VEGF) signaling. Studies show that synthetic coumarins can inhibit tumor angiogenesis and block endothelial cell migration, reducing the metastatic potential [14,38]. In hepatocellular carcinoma models, benzylsulfone–coumarin derivatives effectively impair metastatic processes by inhibiting matrix metalloproteinases (MMPs) and disrupting tumor cell adhesion [99].

Recent work highlights coumarin-based organoselenium compounds, which act as targeted myeloprotective agents while synergizing with chemotherapeutic drugs like carboplatin [100]. The development of hybrid coumarin molecules incorporating sulfonamides and alkyl chains has further improved drug specificity and reduced off-target effects [101].

Numerous in vitro studies have shown that coumarins have a significant effect on the proliferation and survival of various cancer cell lines. Particularly promising results have been obtained in the case of hematological cancer cells (e.g., leukemia), melanoma, breast cancer and colon cancer, where coumarins have shown cytotoxic activity, often synergistic with conventional anticancer drugs (Table 1).

### 4.2. In Vivo Studies—Use in Animal Models

**Figure 6:**
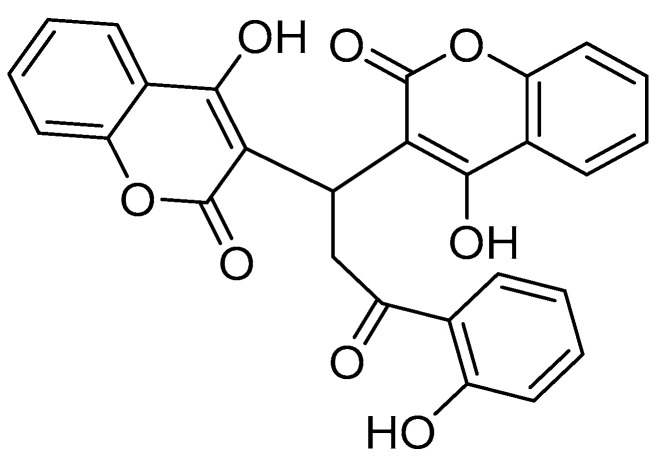
Chemical structure of the synthetic coumarin derivative OT48 {3,3′-[3-(2-hydroxyphenyl)-3-oxopropane-1,1-diyl]bis(4-hydroxy)-2*H*-chromen-2-one}.

The transition from cell-based assays to animal models is a crucial step in preclinical validation. Studies in zebrafish xenografts have demonstrated that umbeliprenin, a naturally occurring sesquiterpene coumarin, significantly reduces the tumor burden and inhibits cell proliferation [118]. In murine breast cancer models, 7-isopentenyloxycoumarin has been found to exhibit potent antiangiogenic activity by modulating CCL2 chemokine signaling, leading to decreased tumor vascularization and growth [119]. Similarly, OT48, a synthetic coumarin derivative, was shown to inhibit tumor progression in spheroid-based models and 3D organoid systems (Figure 6) [120].

Preclinical studies suggest that certain coumarin derivatives enhance immune responses by modulating macrophage polarization and T-cell activation [121]. These findings align with nanoparticle-based coumarin formulations, which improve drug bioavailability while boosting immune-mediated tumor suppression [122]. Moreover, pre-treatment with hydroxylated coumarins has been shown to increase the efficacy of platinum-based chemotherapy in lung and ovarian cancer models, suggesting a role in chemosensitization [123].

The antitumor efficacy of selected natural coumarin derivatives has also been confirmed in in vivo models. Studies conducted on laboratory animals, most often mice with implanted human tumors (xenografts), have shown that some compounds from this group significantly inhibit tumor growth, reduce angiogenesis and induce apoptosis of tumor cells. The therapeutic effects are often associated with the modulation of key signaling pathways (e.g., PI3K/Akt, MAPK, NF-κB), which confirms their potential in targeted therapy (Table 2).

### 4.3. Review of Pharmacokinetic and Bioavailability Studies

Despite their promising anticancer efficacy, many coumarins suffer from low solubility and bioavailability. Studies indicate that lipophilic coumarins demonstrate enhanced oral absorption, whereas highly hydroxylated derivatives often undergo rapid hepatic metabolism via cytochrome P450 enzymes [15]. Recent pharmacokinetic evaluations of coumarin–palladium(II) complexes show improved plasma stability and prolonged circulation time, suggesting enhanced drug retention and efficacy [133].

To overcome the pharmacokinetic limitations, researchers have explored nanocarrier-based delivery methods. Studies show that polymeric nanoparticles and liposomal encapsulation significantly enhance the drug half-life and tumor accumulation [123]. A nanoformulated coumarin hybrid system, developed for pancreatic cancer, demonstrated a three-fold increase in bioavailability and targeted accumulation in tumor tissues, highlighting the potential of nanomedicine-based interventions [122]. Understanding the structure–activity relationships (SARs) of coumarins is crucial to optimizing their pharmacological profiles. A recent QSAR analysis by Sabt et al. showed that electron-donating substitutions at the 7-position significantly improve antiproliferative activity, while halogenated coumarins exhibit superior enzyme inhibition [98].

Additionally, computational docking studies have provided valuable insights into coumarins’ interactions with oncogenic targets, further guiding drug design and optimization [134].

The preclinical evaluation of coumarins has provided strong evidence supporting their anticancer potential. In vitro studies confirm their cytotoxicity, in vivo models validate their efficacy in animal systems, and pharmacokinetic studies reveal essential modifications needed to optimize their clinical translation. However, challenges such as bioavailability limitations, off-target effects, and metabolic instability remain key barriers. Future research should focus on improving drug delivery, enhancing combinatorial approaches, and bridging the gap between preclinical and clinical studies to fully exploit coumarins’ therapeutic potential in oncology.

## 5. Potential Use of Coumarins in Cancer Therapy

### 5.1. Possibilities for Use as Stand-Alone Anticancer Drugs

Coumarins exert direct cytotoxic effects on various cancer cell lines by inducing apoptosis and cell cycle arrest. Several hydroxylated and prenylated derivatives have demonstrated potent antiproliferative activity through the activation of caspases and the mitochondrial apoptotic pathway [87]. For example, daphnetin and esculetin have shown strong activity against melanoma and colorectal cancer by modulating the Bcl-2/Bax ratio and promoting caspase-9 activation [97,135].

Angiogenesis is a key target in cancer therapy, and several coumarin derivatives have exhibited antiangiogenic properties by inhibiting vascular endothelial growth factor (VEGF) signaling [14]. For instance, benzylsulfone–coumarins have been shown to disrupt endothelial tube formation, thereby preventing tumor vascularization and metastasis [99].

Photodynamic therapy (PDT) has emerged as a promising non-invasive cancer treatment approach, utilizing photosensitizing agents that, upon activation by light of a specific wavelength, generate reactive oxygen species (ROS) to induce tumor cell death. Recent advancements in synthetic and naturally derived coumarin-based photosensitizers have demonstrated significant potential in improving the efficacy and selectivity of PDT for various malignancies [86,136]. Coumarins possess intrinsic fluorescence properties that allow their use as light-activated therapeutic agents in PDT. The anticancer activity of coumarin-based photosensitizers relies on three main mechanisms: generation of singlet oxygen (^1^O₂) and ROS, mitochondrial targeting and apoptosis induction and inhibition of tumor angiogenesis.

A study by Ortega-Forte et al. demonstrated that COUPY–coumarin derivatives selectively accumulate in the mitochondria of HeLa cells. Upon light activation, these compounds induced severe mitochondrial dysfunction, leading to rapid ROS production and cell death. In xenograft models, COUPY–coumarins significantly inhibited tumor progression and reduced recurrence rates (Figure 5) [86].

Recent advancements in PDT include coumarin–porphyrin conjugates, which exhibit enhanced tumor selectivity due to their two-photon absorption properties. Wu et al. reported that these hybrid molecules demonstrated about 50% higher ROS generation efficiency compared to conventional porphyrin-based photosensitizers [137]. Studies on furanocoumarins, such as psoralen, show that these naturally occurring compounds possess dual anticancer effects, acting as both chemotherapeutic agents and photosensitizers. In cancer cells, psoralen-based PDT can lead to tumor regression, suggesting a synergistic interaction between photodynamic and cytotoxic effects [14,138].

One of the primary limitations of PDT is the hydrophobic nature of most photosensitizers, which affects their solubility and bioavailability. To overcome this problem, a method for nano-encapsulation of coumarins was developed, which showed improved bioavailability and targeted accumulation in the tumor [139].

A novel strategy involves the design of coumarin hybrids that act as both photosensitizers and chemotherapeutic agents. Ahmadi and Haddadi-Asl synthesized a doxorubicin–coumarin conjugate, which demonstrated synergistic cytotoxicity in cancer cells models when exposed to visible light irradiation [140].

### 5.2. Coumarins as Adjuvants in Combination Therapy with Chemotherapy

Coumarins have demonstrated chemosensitizing effects when used in combination with standard chemotherapeutic agents. For example, pre-treatment with hydroxylated coumarins has been found to enhance the efficacy of platinum-based drugs in lung and ovarian cancer models, increasing tumor cell sensitivity to cisplatin and carboplatin [79].

One of the most significant challenges in chemotherapy is multidrug resistance (MDR). Several coumarins act as P-glycoprotein inhibitors, effectively blocking drug efflux pumps and enhancing intracellular drug retention [137,141,142]. In vitro studies have shown that sesquiterpene coumarins (SCs) can be used as chemosensitizers. Kasaian et al. isolated and purified 14 SCs from the roots of four *Ferula* species. The purified SCs were evaluated for their multidrug resistance (MDR) reversal properties in A2780/RCIS cells (cisplatin-resistant derivatives of the human ovarian cancer cell line A2780P). Among the tested compounds, mogoltacin, mogoltadone, farnesiferol A, farnesiferol B, farnesiferol C, lehmferin, conferdione, and samarcandine showed significant MDR reversal effects. The combination of nontoxic concentrations of SCs (20 μM) with cisplatin significantly enhanced the cytotoxicity of cisplatin on A2780/RCIS cells. The finding showed that conferdione and samarcandine had the strongest inhibitory effect on the efflux of multidrug resistance-associated pump protein 2, and thus these compounds could be considered prime scaffolds for further structural modifications [143].

### 5.3. Pharmaceutical Formulations—Possibilities for Improving Efficacy and Bioavailability

Despite their promising anticancer properties, many coumarins suffer from poor solubility, rapid metabolism, and low bioavailability. Hydroxylated derivatives are often rapidly metabolized by hepatic enzymes, limiting their systemic availability [15].

Recent advances in nanomedicine have led to the development of nanoformulations of coumarins, which significantly enhance the drug half-life and tumor accumulation. Liposomal and polymeric nanoparticles loaded with coumarin derivatives have exhibited improved plasma retention times and increased tumor specificity [144]. A notable example is coumarin-loaded micelles, which increase water solubility and improve intracellular drug uptake, thereby enhancing anticancer efficacy [145]. Hybrid drug formulations combining coumarins with conventional chemotherapeutics have also been explored to improve treatment outcomes. Coumarin–steroid conjugates have been found to exhibit dual anti-inflammatory and anticancer effects, making them suitable for hormone-sensitive tumors such as prostate and breast cancers [82].

Coumarins exhibit remarkable anticancer potential and can be utilized as stand-alone therapeutic agents, adjuvants in chemotherapy, and components of novel pharmaceutical formulations. Their ability to target multiple pathways, enhance drug efficacy, and modulate drug resistance highlights their clinical relevance in oncology. However, challenges related to bioavailability and metabolism must be addressed through nanotechnology and drug formulation advancements. Future research should focus on optimizing coumarin derivatives, identifying novel molecular targets, and conducting large-scale clinical trials to fully establish their therapeutic utility in cancer treatment.

## 6. Safety and Limitations of Coumarins in Cancer Therapy

### 6.1. Potential Toxicity and Side Effects

Coumarins, despite their promising anticancer properties, exhibit potential toxicity risks that must be carefully evaluated before clinical applications. Their adverse effects are mainly associated with hepatotoxicity, nephrotoxicity, and hematological disturbances [15].

One of the most documented safety concerns regarding coumarins is their potential to cause hepatic damage, particularly in high doses or after prolonged use [87]. Some naturally occurring coumarins, such as psoralen and umbeliferon, have been linked to elevated liver enzymes, fatty liver disease, and hepatocellular necrosis Animal studies suggest that chronic exposure can lead to oxidative stress-mediated hepatic injury, limiting their long-term use in therapy [146].

Studies on zebrafish models have shown that some coumarins disrupt kidney function by interfering with renal transporters, leading to electrolyte imbalances and nephrotoxicity [121]. Moreover, some derivatives have demonstrated hematological toxicity, such as leukopenia and thrombocytopenia, raising concerns regarding their safe therapeutic window in oncology [147].

Coumarins like furanocoumarins exhibit phototoxic effects, making patients susceptible to UV-induced skin damage. This reaction is particularly problematic for individuals undergoing photodynamic therapy (PDT), as excessive exposure can trigger erythema, hyperpigmentation, and DNA damage [148].

### 6.2. Interactions with Other Anticancer Drugs

While coumarins have shown synergistic effects with conventional chemotherapy, they can also interfere with drug metabolism, posing potential risks.

Coumarins have been reported to enhance or inhibit the efficacy of chemotherapy drugs by modulating drug transporters such as P-glycoprotein (P-gp) [8,149]. For instance, warfarin-like coumarins can inhibit CYP450 enzymes, leading to altered drug metabolism and enhanced toxicity [150]. Additionally, combining coumarins with platinum-based chemotherapy has been found to increase nephrotoxicity, necessitating careful dose adjustments [137].

Some coumarin derivatives act as sensitizers in relation to targeted therapy by inhibiting angiogenesis and tumor progression [49]. Curcumin, for example, enhances sorafenib activity in hepatocellular carcinoma models by modulating VEGF and MAPK signaling pathways [151]. However, in some cases, these interactions increase the risk of endothelial dysfunction and bleeding complications [79].

Coumarins can also influence immune checkpoint inhibitors by altering cytokine production and T-cell responses. While this may boost anticancer immune responses, it also raises concerns regarding immune-related adverse effects such as autoimmune hepatitis and colitis [45,79].

### 6.3. Limitations of Use in Patients with Comorbidities

The potential clinical utility of coumarins in cancer therapy is further limited by pre-existing health conditions, which can exacerbate adverse effects and complicate treatment regimens.

Coumarins, particularly anticoagulant derivatives, have been associated with bleeding risks and thrombocytopenia, making them unsuitable for patients with cardiovascular diseases [152]. Their warfarin-like effects can lead to hemorrhagic complications, especially in patients undergoing chemotherapy with agents like 5-fluorouracil or gemcitabine [153,154].

Patients with pre-existing liver disease may experience enhanced hepatotoxicity, limiting the safe use of esculetin- and psoralen-based coumarins [155]. Similarly, renal impairment patients may be at greater risk of coumarin-induced nephropathy, particularly when combined with nephrotoxic agents such as cisplatin or methotrexate [156].

Diabetic patients may also be at risk, as some coumarins modulate glucose metabolism, potentially leading to hypoglycemia when co-administered with metformin or insulin [157]. Moreover, due to their extensive hepatic metabolism, coumarins require careful monitoring in patients with obesity or metabolic syndrome [158].

While coumarins present exciting opportunities in anticancer therapy, their safety limitations must be critically addressed before widespread clinical adoption. The key concerns include hepatotoxicity, nephrotoxicity, and drug interactions, which pose risks in patients with comorbidities. Strategies such as dose optimization, prodrug formulations, and combination therapy adjustments are necessary to mitigate toxicity and improve their therapeutic index. Future research should focus on large-scale clinical trials to fully assess the long-term safety profiles and develop novel coumarin derivatives with reduced toxicity and enhanced selectivity.

## 7. Research Perspectives and Future Development Directions

Coumarins, due to their structural diversity and pharmacological activity, are promising candidates for new anticancer drugs. Research on their synthetic derivatives and their use in targeted therapies, as well as technologies for improving their bioavailability, opens up new perspectives in cancer treatment (Table 3).

### 7.1. New Synthetic Coumarin Derivatives—Directions for Drug Design

New synthetic coumarins are designed to have enhanced anticancer activity by modifying the benzopyrone ring and adding functional groups that increase the potency of their interactions with target proteins [15]. For example, halogenated coumarins show stronger inhibition of tyrosine kinases, which are key to tumor growth [14], pyrazole-conjugated coumarins show high efficacy in inhibiting melanoma cell proliferation [162], and coumarin hybrids containing sulfonamide groups are more selective for estrogen receptors in breast cancer [163].

Synthetic coumarins act in multiple ways, including by inhibiting angiogenesis caused by blocking the VEGF and HIF-1α pathways [14], by inducing oxidative stress and activating apoptotic pathways [35], and by influencing epigenetics—studies indicate that coumarins can inhibit histone acetylation, which leads to the suppression of oncogene expression [164].

### 7.2. Application of Nanotechnology to Improve the Efficacy of Coumarins

One of the main limitations of coumarins is their low bioavailability and rapid hepatic metabolism. The use of nanotechnology allows for the improvement of their stability and targeted delivery to cancer cells [165]. Modern nanotechnological strategies for coumarins are liposomal delivery systems—they improve the half-life and penetration of solid tumors [166]—and polymeric nanoparticles—the biophysical characteristics of the nanoformulation, combined with its selective absorption by cancer cells, low in vivo toxicity profile and effective action on tumors, prove the versatility and potential of this nanoparticle formulation in drug delivery applications [167]. Coumarins conjugated with gold nanoparticles show increased absorption by cancer cells and a photosensitizing effect in photodynamic therapy [168].

### 7.3. Potential of Coumarins in Personalized Therapy

Personalized anticancer therapy is based on adapting the treatment to the genetic profile of the patient, which allows for greater efficacy and fewer side effects. Coumarins show potential in this field, especially due to their ability to interact with key signaling pathways and influence the expression of cancer genes [79].

Studies indicate that some coumarins can act as HDAC (histone deacetylase) inhibitors and modulators of DNA methylation, which allows for the regulation of the expression of genes associated with the development of cancer [169]. Coumarins in combination with immune checkpoint inhibitors (e.g., anti-PD1) can increase the effectiveness of immunotherapy [170].

Pharmacogenetics of coumarins—studies on their metabolism can help in selecting the optimal dose for patients by estimating the therapeutic dose of coumarin, while therapy based on pharmacogenetics can improve the safety and efficacy of coumarin therapy [171].

In the coming years, large-scale clinical trials will be crucial to confirm the therapeutic viability of coumarins in human patients. Their potential application in combination therapies, immunotherapy, and precision medicine requires further validation through comprehensive clinical research. If these challenges can be addressed, coumarins may soon become an integral component of modern oncological treatments, offering novel therapeutic strategies for drug-resistant and hard-to-treat cancers.

## 8. Conclusions

Coumarins have emerged as a promising class of compounds in anticancer therapy, demonstrating diverse mechanisms of action, including apoptosis induction, inhibition of tumor proliferation, modulation of oxidative stress, and suppression of angiogenesis and metastasis. Their ability to interfere with multiple signaling pathways, such as the PI3K/Akt/mTOR, Wnt/β-catenin, and NF-κB pathways, underpins their therapeutic potential. Additionally, synthetic coumarin derivatives have shown improved selectivity and efficacy, opening up avenues for their development as next-generation anticancer drugs.

Despite these promising results, several challenges hinder the clinical translation of coumarins. Bioavailability issues, such as poor solubility and rapid hepatic metabolism, limit their systemic exposure and therapeutic efficacy. Advances in nanotechnology, including liposomal formulations, polymeric nanoparticles, and targeted drug delivery systems, are crucial to overcoming these limitations and ensuring optimal drug concentrations at tumor sites. Additionally, coumarins’ interactions with chemotherapeutics and their potential role as chemosensitizers provide opportunities for combination therapy, enhancing treatment efficacy while reducing drug resistance.

Safety concerns, including hepatotoxicity, nephrotoxicity, and interactions with other anticancer drugs, necessitate further investigation. Careful dose optimization and pharmacogenetic studies may help minimize these risks, ensuring patient safety and treatment success. Furthermore, the individualized application of coumarins in precision medicine, tailored to tumor-specific genetic and epigenetic profiles, represents a significant step toward personalized cancer therapy.

Looking ahead, future research should focus on the rational design of new coumarin derivatives, advanced formulation strategies, and large-scale clinical trials to validate their efficacy and safety in human patients. Expanding our understanding of coumarin-based pharmacology through molecular modeling, biomarker identification, and drug repurposing approaches will be key to unlocking their full potential in modern oncology.

In summary, coumarins represent a versatile and promising group of anticancer agents, with broad mechanistic capabilities and clinical relevance. While challenges remain, ongoing research efforts in synthetic drug design, nanomedicine, and personalized medicine will play a crucial role in harnessing their full therapeutic potential, paving the way for their integration into next-generation cancer treatment regimens.

## Figures and Tables

**Table 1:** Anticancer efficacy of selected natural coumarin derivatives in vitro.

Coumarin	Cell Lines	Disease	IC_50_	Mechanism	Ref.
Imperatorin	HepG2	Liver cancer	60.58 µM	Apoptosis is induced through mitochondrial dysfunction, activation of caspase cascades, proteolytic cleavage of PARP, and DNA fragmentation	[102]
Isoimperatorin	SGC-7901	Gastric adenocarcinoma	18.75 µg/mL	The compound targets critical proteins involved in the intrinsic apoptotic pathway, such as Survivin, Mcl-1, Bcl-xL, Bcl-2, Smac, Bax, cleaved caspase-3, and caspase-9	[103]
Scopoletin	CCRF-CEM	Lymphoblastic leukemia	2.6 µM	It inhibits cell proliferation and overcomes multidrug resistance in leukemia cells	[104]
Umbeliprenin	AGS BGC-823	Gastric adenocarcinoma Gastric cancer	11.74 µM 24.62 µM	Inhibits gastric cancer cell migration via downregulation of Wnt/β-catenin and MMP pathways	[105]
Isopimpinellin	Saos-2 HOS HT-29 SW620 RPMI8226, U266	Metastatic osteosarcoma Metastatic colorectal adenocarcinoma Multiple myeloma	42.59 µM 321.6 µM 95.53 µM 711.3 µM 105.0 µM 84.14 µM	It induces apoptosis through activation of caspase-3 and inhibition of DNA synthesis	[106]
Auraptene	MCF-7	Breast cancer	61.3 µg/mL	Induces apoptosis through activation of caspase-3 and caspase-8, while concurrently inhibiting MMPs, VEGFRs, and angiogenesis	[107]
Daphnetin	Human melanoma		40.48 µM	Enhances mitoxantrone’s cytotoxicity	[96]
Fraxetin	J82 cells	Urinary carcinoma		Inhibits the Akt pathway	[108]
Ellagic acid *	PANC-1	Pancreas carcinoma	7.38 µg/mL	Inhibits pancreatic cancer by inducing G1 cell cycle arrest, COX-2 suppression, and EMT reversal	[109]
	U87 U118	Glioblastoma Glioblastoma	91.2 µM 98.6 µM	Inhibits the Akt and Notch signaling pathways	[110]
Meliquercifolin A	HeLa P-388	Cervical cancer Murine leukemia	2.6 µM 78.9 µM	Cytotoxic	[111]
Beccamarin	Raji HeLa	Burkitt lymphoma Uterus adenocarcinoma	2.92 µg/mL 3.91 µg/mL	Cytotoxic	[112]
Neotanshinlactone	MCF-7 ZR-75-1 SK-BR-3	Breast cancer Breast cancer Breast adenocarcinoma	0.6 µg/mL 0.3 µg/mL 0.2 µg/mL	Selectively inhibits estrogen receptor-positive breast cancer cells by transcriptional down-regulation of ERα and inducing apoptosis	[113,114]
Penisarins B	HL-60 A-549 MCF-7 SW480	Leukemia Lung Carcinoma Breast cancer Colon Adenocarcinoma	3.6 µM 15.3 µM 13.0 µM 27.1 µM	Cytotoxicity	[115]
Ferulin C	MCF-7 MDA-MB-231	Breast adenocarcinoma	19.8 µM 15.6 µM	Inhibition of tubulin polymerization	[116]
Calanone	HeLa	Uterus adenocarcinoma	22.87 µg/mL	Induces apoptosis by increasing p53 expression	[117]

* Structurally, ellagic acid (EA) is a dilactone of hexahydroxydiphenic acid (HHDP), a dimeric gallic acid derivative, produced mainly by the hydrolysis of ellagitannins, a widely distributed group of secondary metabolites.

**Table 2:** Anticancer efficacy of selected natural coumarin derivatives in vivo.

Coumarin	Animal Model	Injected Cells	Dose/ Duration	Disease	Mechanism	Ref.
Auraptene	Xenograft mouse	SRB12-p9 cells	1000 ppm auraptene+ 10 ppm retinoic acid/ 7 days	Human skin cancer	Inhibition of the Stat3 and NF-κB pathways	[124]
Daphnetin	Dawley rats	HUVECs	150 µM	Angiogenesis	Inhibits NF-κB and VEGF pathways, inhibits angiogenesis, and induces apoptosis	[125]
Fraxetin	Xenograft mouse	J82 cells	20 mg/kg/ 28 days	Bladder cancer	Inhibition of the Akt pathway	[108]
Ellagic acid *	Mice	PANC-1 cells	40 mg/kg/ 14 days	Pancreatic cancer	Reduces G1 arrest, and suppresses NF- κB, COX-2, MMPs, and Akt	[109]
		HOP62 cells	40 mg/kg/ 22 days	Lung cancer	Induces autophagy-mediated cell death by inhibiting mTOR, reducing CIP2A	[126]
		U87 cells	40 mg/kg/ 28 days	Glioblastoma	Upregulates E-cadherin, downregulates Snail, MMPs, inhibits Akt signaling	[110]
Herniarin	Rats	Anthracene- induced	20 mg/kg/ 28 days	Breast cancer	Modulates gene expression via the LXR/PI3K/Akt/Maf1 pathway	[127]
Ferulin C	Mice	MCF-7 cells	25 mg/kg/ 16 days	Breast cancer	Induces G1/S arrest, apoptosis, and autophagy, inhibits Ras-Raf-ERK, AKT- mTOR pathways.	[116]
Osthole	Mice	Y-79 cells	0.5 mmol/kg/ 36 days	Retinoblastoma	Decreases p-PI3K/PI3K, p-AKT/AKT, and p-mTOR/mTOR ratios	[128]
Imperatorin	Mice	HepG2 cells	100 mg/kg/ 14 days	Hepatocellular carcinoma	Apoptosis, mitochondrial dysfunction, caspase activation	[102]
Scopoletin	Mice	HCT-116 cells	100 mg/kg/ 7 days	Human tumor vascularization	ERK1, VEGF-A, FGF-2 modulation	[129]
Umbeliprenin	Mice	4T1 cells	200 µL/day/ 18 days	Breast cancer	Inhibits 4T1 tumor growth by ↓ angiogenesis, metastasis, inflammation; ↑ antitumor immunity	[130]
		BGC823	10 mg/kg/ 12 days	Gastric cancer	Inhibits tumor migration by blocking MMPs, GSK-3β, and Wnt signaling	[105]
		LLC cells	2.5 mg/200 mL	Lung cancer	Shifts Th1/Th2 balance toward Th1, reduces tumor size, ↑TNF-α/IFN-γ, ↓Foxp3, TGF-β, IL-10, IL-4	[131]
Isopimpinellin	Zebrafish embryos			Antiangiogenic effects	Inhibits angiogenesis via VEGF/AKT/HIF-1α suppression and miR-15b-5p/miR-542-3p regulation	[132]

* Structurally, ellagic acid (EA) is a dilactone of hexahydroxydiphenic acid (HHDP), a dimeric gallic acid derivative, produced mainly by the hydrolysis of ellagitannins, a widely distributed group of secondary metabolites.

**Table 3:** Promising anticancer efficacy of selected coumarin derivatives in vivo.

Coumarin	Animal Models	Injected Cancer Cells	Dose/ Duration	Disease	Mechanism	Ref.
Isoimperatorin	Mice	SGC-7901	10 mg/kg/ 20 days	Gastric adenocarcinoma	Apoptosis induction, BAX↑, Survivin↓	[103]
Osthole	Mice	MDA231BO	5.25 mg/kg/42 days	Breast cancer	Inhibits TGF-β/Smad signaling and breast cancer bone metastasis	[159]
		HCCC-9810	50–100 mg/kg/ 25 days	Intrahepatic cholangiocarcinoma	PI3K/Akt pathway	[21]
Anticarin-β	Mice	U87-MG	1 mg/kg/ 3 days	Glioma	Induces apoptosis via STAT3, Akt, and MAPK pathways	[160]
		MG63-LUC	5 mg/kg/ 49 days	Osteosarcoma	Inhibits CCT4, disrupts proteostasis, and suppresses osteosarcoma growth	[161]
